# 24. FROM DUSK TILL DAWN: LIFELONG TRAJECTORIES OF COGNITIVE FUNCTIONING IN PSYCHOTIC DISORDERS AND THEIR IMPLICATIONS FOR FUNCTIONAL RECOVERY AND TREATMENT DECISION

**DOI:** 10.1093/schbul/sby014.095

**Published:** 2018-04-01

**Authors:** Eva Velthorst

**Affiliations:** Icahn School of Medicine at Mount Sinai

## Abstract

**Overall Abstract:** This symposium will draw together state of the art findings on the lifelong cognitive trajectories, on key-predictors of cognitive functioning and the functional consequences of cognitive impairments in schizophrenia and related psychotic disorders from developmental epidemiological, prodromal, and clinical research. Four speakers will take the audience through new findings on the cognitive course of the lifespan, ranging from childhood to old age. Specifically, the talks will address four key-questions:

1) Which areas of cognitive functioning are impaired and when does this impairment start?

2) How well can cognitive functioning predict the development of a psychotic illness, as well as diagnostic and functional outcome?

3) Does cognitive functioning remain stable after illness onset or are psychotic disorders characterized by continuing decline? When does decline occur and is it possible to predict it?

4) And what is the functional sequelae of specific cognitive impairments in older adults with schizophrenia?

Specifically, Dr. Mollon will present new data examining the origin of cognitive impairment across the psychosis spectrum using a population-based cohort followed prospectively from birth. Her findings demonstrate that while individuals with affective psychosis, subthreshold psychotic experiences and even depression experience some degree of cognitive impairment across the first two decades of life, only those who go on to develop non-affective psychosis exhibit large, widespread and increasing deficits.

Most studies of neurocognitive functioning in Clinical High Risk (CHR) cohorts have examined group averages, likely concealing heterogeneous subgroups. The study of Dr. Velthorst therefore used two independent methods to identify neurocognitive subgroups in a large population at Clinical High Risk for developing psychosis. Her findings show that neurocognitive profiles vary substantially in their severity and are associated with diagnostic and functional outcome, underscoring neurocognition as a predictor of illness outcomes.

Dr. Fett will present recent research on cognitive functioning in a large sample of patients at first hospitalization for a psychotic disorder who have been followed 20-years into the illness. Her findings indicate that cognitive functioning in psychotic disorders continues to decline after illness onset, that this decline is not specific to schizophrenia but present across psychotic disorders, and that, relative to never-psychotic individuals, impairments on some key-cognitive domains worsen with age. Decline could not reliably be predicted by key patient characteristics at baseline.

Lastly, Dr. Harvey will share novel data on the course of cognitive functioning in middle aged and older patients with schizophrenia. His findings demonstrate that cognitive impairments are moderated in their impact on everyday outcomes by the presence of severe communication abnormalities. Interestingly, verbal under-productivity and disconnected speech had different functional correlates, with under-productivity impacting clinician rated social outcomes and performance on measures of interpersonal social competence.

A lifetime focus on cognition is paramount in order pinpoint critical periods for prevention and intervention. This symposium seeks to present a comprehensive overview of the cognitive landscape of psychotic disorders by integrating findings on predictors and consequences of lifelong cognitive functioning of individuals diagnosed with a psychotic disorder.

